# Optimization studies of stir casting parameters and mechanical properties of TiO_2_ reinforced Al 7075 composite using response surface methodology

**DOI:** 10.1038/s41598-021-99168-1

**Published:** 2021-10-06

**Authors:** Adeolu A. Adediran, Abayomi A. Akinwande, Oluwatosin A. Balogun, Bayode J. Olorunfemi, Saravana Kumar M.

**Affiliations:** 1 Materials Design and Structural Integrity Group, Department of Mechanical Engineering, Landmark University, Omu-Aran, Kwara State Nigeria; 2 Department of Metallurgical and Materials Engineering, Federal University of Technology, Akure, Ondo State Nigeria; 3 Department of Mechanical Engineering, Federal University Oye-Ekiti, Oye-Ekiti, Ekiti State Nigeria; 4 Department of Production Engineering, National Institute of Technology, Tiruchirappalli, Tamil Nadu India

**Keywords:** Engineering, Materials science

## Abstract

Stir casting is a common metallurgical route in the casting of aluminum composites. Series of work done in this aspect considered the development of the composites with fixed stir casting parameters without applying an optimization approach. These parameters affect the microstructure and performance of the composites. The study is focused on the optimization of the stir casting parameters in the production of Al 7075 reinforced with TiO_2_ microparticles for performance improvement. Three stir casting parameters of stirring temperature, speed, and time were varied and optimized using the central composite design technique of the response surface method. Properties evaluated were ultimate tensile strength, hardness, impact strength, elastic modulus, and compressive strength. ANOVA results showed that the three stir casting parameters had a significant impact on the property responses. Five quadratic models were established for the properties linking them to the factors. The models were confirmed to be statistically significant at a confidence level of 95% and variations were observed to be < 5%. The interaction profile of the parameters as per response surface was analyzed. Contour plots associated with each interaction gave different ranges of stirring parameters in which each property can be maximized. Simultaneous optimization of the properties using Minitab 19 software showcased 779.3 °C, 574.2 rpm, and 22.5 min as the optimal stir casting parameters for temperature, speed and time respectively.

## Introduction

Ceramic particle reinforcement of aluminum alloy matrix is a common practice in engineering, in which the developed materials are used for many applications^1^. Owing to the versatility of aluminum alloy in modern day engineering applications, a series of works has been geared at the development of high-performance aluminum composites. The high strength to weight ratio possessed by the alloy and its composite gives it an advantage over steel for applications involving light weight and fuel efficiency hence, used in automobile and aerospace applications. Al 7075-T6 is one of the alloys in the 7000 aluminum alloy series and possesses good mechanical properties and fit for many applications. Recent researches have been geared to improving properties of the alloy by addition of ceramic nano and micro particles, one of which is titanium-based ceramic particles^2–5^. Titanium based particulates has a wide range of applications as reinforcement particles in metal matrix. Findings from Ravi ^6^ reported on the reinforcement of Al 7075 matrix with SiC and TiC. The particles were added via stir casting production route in the proportion of 5% SiC, 5% TiC and the mix of 5% SiC and 5% TiC. It was observed that the yield, ultimate strength and impact strength were the highest when the mix of 5% SiC and 5% TiC was incorporated. In Kumar et al.’s work, Al 7075 was reinforced with TiC at varying proportions of 3, 4, 5, 6, and 7%^7^. It was observed that the presence of particulate enhanced the microhardness, yield strength, and ultimate tensile strength as the proportion increased to 7 wt%. However, at 1.5, 3, 4.5, and 6 wt% TiB_2_ was infused in Al 7075 as observed by Ramkumar et al^8^, this led to enhancement of hardness and bending strength. Their study revealed that the microstructure showed a particle dispersion within the matrix, thereby enhancing the properties. It was reported that titanium boride dispersion in Al 7075 matrix resulted in the enhancement of tensile strength and hardness at particulate dosage of 4, 8, and 12 wt%^9^. Infusion of TiC in Al 7075 led to the improvement of yield and tensile strength even as optimum enhancement was attained at 9 wt% TiC inclusion. Similar trend was achieved when Kumar et al^7^ added Titanium carbide at 3, 4, 5, 6, and 7 wt% of the Al matrix. Microhardness, yield, and ultimate tensile strength were enhanced as the particulates increased in the stir cast product. Optimum enhancement of strength was achieved at 7 wt% content. The choice of TiO_2_ in reinforcing and enhancing properties of Al 7075 was taken by Murali et al^10^, the compressive and tensile strength were enhanced with titanium dioxide addition even as the optimum enhancement was attained at 15 wt%. Comparative study showed that titanium dioxide proportions of 1, 3, 5, 7, and 9 wt% led to a progressive and linear increase in yield and ultimate tensile strength while microstructural examination showed even dispersion of particles within the matrix^11^. Al 6061 was reinforced with 50 µm average sized titanium dioxide, as investigated by Kumar et al^12^. Evaluation of the properties showed that relative to the base metal, the hardness was improved by 20.7, 52.6, and 66.7% and the ultimate tensile strength was enhanced by 31.6, 55.8, and 89.5% as the particulate increased at 1, 2, and 3 wt%. It is concluded that the titanium dioxide particles reinforced composite exhibited improved properties compared to the base metal. Authors Alagarsamy and Ravichandran^13^ employed the stir casting route in the development of TiO_2_–AA 7075 composites with varying proportions of 5, 10, and 15 wt% titanium dioxide particles. The mechanical properties; tensile impact, flexural and compressive strengths, and hardness were enhanced as the particulate increased up to 10 wt%. Authors Kumar et al^14^ and Kumar et al ^15^have performed the investigation of the influence of the stir casting parameters on the distribution of the reinforcement particles using the visualization technique. From their investigation, it was concluded that 40% impeller position from the base 45º blade angle shows the considerable improvement in the mechanical properties. Computational Fluid Dynamics simulations were employed to evaluate the effect of vortex pressure, which was created during the stirring action, on the processing of the MMC. The results concluded that the mechanical properties were improved by the optimal vortex height using Taguchi design of the experiment^16^. Kumar et al^17^ also performed the microstructural evaluation to identify the influence of stir casting parameters on the mechanical properties of the composites. The microstructural results show a uniform dispersion of reinforcement particles which consecutively increases the mechanical properties of the composites. Even though various techniques were involved in the evaluation of the stir casting process parameters, no one has performed the RSM optimization technique to determine the best stir casting parameters for the processing of Metal Matrix Composites. The afore-discussed literature centered on the effect of composition on the properties of Al 7075 without considering the effect of varying stir casting process parameters like stirring temperature, time, and speed on properties of the alloy. Other process parameters are blade angle, stirrer height, feed rate of reinforcement, and direction of impeller rotation. These is important to ascertain how these parameters affect the microstructure and optimize for the most appropriate conditions for improvement of the alloy. This research reveals the effect of stirring parameters; temperature, time, and speed on the ultimate tensile strength, microhardness, impact strength, elastic modulus, and compressive strength of Al 7075/10 wt% microparticles of titanium dioxide (average size 13 µm) according to the study of Alagarsamy and Ravichandran^13^ and El-Mahallawi et al ^18^ in which 10 wt% yielded the optimum performance.

## Methodology

### Material preparation

Aluminum alloy ingot AA 7075-T6 was employed in this study. The chemical composition as obtained via spectrometer test is highlighted in Table 1 while the properties are stated in Table 2. Stir casting process was done in a graphite crucible (diameter 100 mm and height 175 mm). Titanium dioxide (TiO_2_) microparticle of size 13 µm was preheated to 500 °C for 10 min and was introduced into the melt at 10 wt%. The range of stirring speed, temperature, and time considered are presented in Table 3. The specimens were prepared in accordance with the experimental runs obtained via the central composite design as stated in Table 4. To enhance the wettability of matrix, 1 wt% magnesium was added into the melt as previously reported by Kumar et al^19^.

**Table 1:** Elemental composition of Al 7075 alloy used in the study.

Element	Zn	Mg	Cu	Si	Fe	Ti	Cr	Al
Amount (%)	5.9	2.3	1.3	0.2	0.3	0.08	0.04	Balance

**Table 2:** Properties of Al 7075 metal.

Properties	Ultimate tensile strength	Poisson ratio	Relative density
Value	521 MPa	0.33	2.699

**Table 3:** Levels of factors in the design of experiment.

Factors	Low level	Medium level	High level
Stirring temperature (A)	600	700	800
Stirring speed (B)	400	500	600
Stirring time (C)	10	15	20

**Table 4:** Experimental runs, coded level, and responses.

Experimental runs	Coded levels			Factors			Responses				
	A (°C)	B (rpm)	C (min)	A (°C)	B (rpm)	C (min)	YS	UTS	EM	EL	IM
1	− 1	− 1	− 1	700.000	500.000	15.0000	501	581	82.6	8.76	4.21
2	0	0	− 1.68	700.000	500.000	23.4090	525	642	90.1	7.84	5.31
3	0	0	0	700.000	668.179	15.0000	529	689	91.4	7.51	5.74
4	0	0	0	700.000	500.000	15.0000	546	635	86.5	7.52	5.46
5	0	0	0	600.000	600.000	10.0000	575	719	95.3	6.79	6.41
6	0	1.68	0	800.000	600.000	10.0000	588	682	98.2	6.43	6.86
7	1	1	− 1	700.000	500.000	15.0000	522	600	79.4	7.82	4.86
8	− 1	1	1	700.000	500.000	15.0000	559	622	88.6	7.99	5.51
9	− 1.68	0	0	700.000	500.000	15.0000	574	637	92.1	6.62	5.90
10	1.68	0	0	700.000	331.821	15.0000	530	634	87.3	8.55	5.17
11	0	0	0	800.000	600.000	20.0000	558	715	92.3	7.24	5.86
12	1	− 1	1	868.179	500.000	15.0000	564	734	98.5	7.12	6.13
13	0	− 1.68	0	600.000	400.000	10.0000	579	672	92.6	8.08	5.78
14	− 1	− 1	1	600.000	600.000	20.0000	601	745	96.5	7.34	6.84
15	0	0	0	700.000	500.000	6.5910	629	771	102.3	6.09	7.15
16	1	1	1	800.000	400.000	20.0000	561	628	86.7	8.47	5.00
17	0	0	1.68	800.000	400.000	10.0000	582	665	91.2	7.12	5.66
18	− 1	1	− 1	600.000	400.000	20.0000	618	692	93.2	6.83	6.16
19	1	− 1	− 1	700.000	500.000	15.0000	524	600	84.5	8.17	4.24
20	0	0	0	531.821	500.000	15.0000	531	657	86.8	6.94	4.87

YS is the yield strength (MPa), UTS is the ultimate tensile strength (MPa), EM is elastic modulus (MPa), EL is elongation (%), and IM is impact strength (MPa).

### Test procedure

Ultimate tensile strength is the optimum stress in which the given material can tolerate under applied tension without breaking. Machined specimens whose dimensions are 30 mm in length and 5 mm in diameter were tested for tensile strength applying a universal testing machine (Instron 3369 Series). In line with ASTM E 8/E8M-21^20^, a load of 10 kN was applied at a rate of 10^−4^/s and a cross head speed of 3.0 mm/min’. As prescribed by ASTM E09-9^21^, the compressive strength of the samples was examined using the universal testing machine applying a load of 100 kN at a cross speed of 1 mm/min. Vickers microhardness test was done on the specimens in accordance with ASTM E 384-17^22^ on the surfaces of the samples applying a load of 10 N for 10 s on each sample. Impact toughness was also probed subjecting a specimen 10 × 10 mm^2^ initially notched at 45° to high strain impact with the use of pendulum of 300 N in weight while measuring the absorbed energy to failure (ASTM E-23)^23^. In accordance with ASTM E 384-17^24^, field emission scanning electron microscope (JSM-7610 E) was used in accessing the microstructure of the developed samples. From Table 1, the compositional elements of Al 7075 are displayed, Zn and Mg were observed to be present in considerable amounts while Ti and Cr occurred in trace proportions. Table 2 highlights the properties of the base material with the ultimate tensile strength of 521 MPa and relative density of 2.699.

### Design of experiment

Experimental process involves the design of an experiment via the response surface method (RSM) in which the process parameters are optimized. The variables are stirring temperature (A), stirring speed (B), and stirring time (C). RSM utilizes mathematical and statistical means in analyzing the relationships between process parameters and the response parameters. This process has been employed in several literatures for optimization and the outcome showed that experimental runs in the laboratory can be minimized via this process^25–29^. With the aid of Minitab 19 software, a central composite design was employed involving five-level-three-factor. Twenty (20) experimental runs were undergone for each property evaluated, entailing 6 axial runs, 8 factorial runs, and 6 replicates at the center point as carried out in previous studies^22,30^. The second-order polynomial expression in Eq. (1) was employed in accessing the relationship between the process variables and predicted responses.

1 $${\text{Z}} = {\text{A}} + \mathop \sum \limits_{i = 0}^{n} {\text{BX}} + \mathop \sum \limits_{i = 0}^{n} {\text{CX}}2 + \mathop \sum \limits_{ij = 0}^{n} {\text{DXiXj}} + {\text{E}}$$

Z is the predicted response, A is the intercept, B is a linear coefficient for first-order expression, C is a quadratic coefficient for the second-order expression, D is the coefficient of the interaction effect, and E is the random error. First order polynomial model is expressed in Eq. (2).

2 $${\text{Z}} = {\text{A}} + {\text{BX}}_{1} + {\text{CX}}_{2} + {\text{C}}_{{\text{n}}} {\text{X}}_{{\text{n}}} + {\text{E}}$$

where X_1_, X_2_ … X_n_ are the process variables, A is the constant, and Cn is linear of the nth factor constant, while E is the error.

Accuracy of the models were verified by the predicted values of the coefficient of correlation for predicted and adjustable data, relative deviation, root mean square, and mean square error. In the design, temperature was selected between 600 and 800 °C, time between 10 and 20 min, and speed between 400 and 600 rpm as observed in Tables 3 and 4 in accordance with Aynalem^31^.

## Results and discussion

### Analysis of variance and regression models

As highlighted in Table 5, the *p* values for the process variables stirring temperature (A), stirring speed (B), and stirring time (C) are less than 0.05, which reflect the significance of these variables as they determine magnitude of the response. Squared interactions A*A and C*C are also statistically significant, whereas B*B is insignificant. Two-way interaction A*B, A*C, and B*C are significant since *p* > 0.05. Meanwhile, the contribution of parameters for linear variables A, B, and C are 36.5, 9.03, and 27.89%, implication of which shows the order of significance of the variables is in descending order of stirring temperature, stirring speed and stirring time.

**Table 5:** ANOVA on ultimate tensile strength (UTS).

Source	DF	Seq SS	Contribution (%)	Adj SS	Adj MS	F value	*P* value
A	1	8275.8	36.50	8363.6	8363.56	230.59	0.000
B	1	2969.2	9.03	2969.2	2969.19	119.72	0.000
C	1	7911.6	27.89	7911.6	7911.57	235.42	0.000
A*A	1	4173.4	14.27	4173.4	4173.40	132.35	0.000
B*B	1	611.6	2.23	611.6	611.59	16.86	0.111
C*C	1	1833.6	5.87	1833.4	1833.35	133.26	0.000
A*B	1	460.1	1.68	468.5	468.46	12.92	0.065
A*C	1	9.6	0.03	9.6	9.59	0.26	0.614
B*C	1	70.1	0.26	70.1	70.08	1.93	0.182
Error	17	616.6	2.25	616.6	36.27		
Total	26	27,453.4	100.00				

Second order polynomial model obtained for the ultimate tensile strength which incorporates the input variable is expressed in Eq. (3).

3 $$\begin{aligned} {\text{UTS}} & = - 230 + 6.15 A + 0.4908 B + 0.4657 C - 0.1941 A*A \\ & \quad - 0.000405B*B - 0.002861 C*C + 0.002286 A*B \\ & \quad + 0.00086 A*C - 0.00081 B*C \\ \end{aligned}$$

UTS is the ultimate tensile strength, A is the stirring temperature, B is the stirring speed, and C is the stirring time.

From Table 6, the *p* values for the linear terms A, B, and C are less than 0.05, hence, are significant with contributions of 39.58, 20.81, and 29.87% respectively. This implies that the input variables have a significant effect on the hardness response of the properties as the input parameter varies. The square terms A*A, B*B, and C*C are insignificant likewise cross interactions A*B, A*C, and B*C. From the contributions of the linear terms, the stirring temperature has the highest contribution to the response while the stirring time is the next. Stirring speed showed the least contribution amongst the three parameters.

**Table 6:** ANOVA on hardness (Hd).

Source	DF	Seq SS	Contribution (%)	Adj SS	Adj MS	F-Value	P-Value
A	1	8595.3	39.58	8595.3	8595.25	299.38	0.000
B	1	4670.0	20.81	4670.0	4670.03	223.49	0.000
C	1	6561.3	29.87	6561.3	6561.25	258.62	0.000
A*A	1	228.8	1.04	228.8	228.77	9.02	0.074
B*B	1	2.6	0.01	2.6	2.64	0.10	0.748
C*C	1	78.8	0.36	78.8	78.77	3.10	0.584
A*B	1	168.4	0.77	168.4	168.35	6.64	0.089
A*C	1	193.9	0.88	193.9	193.91	7.64	0.063
B*C	1	96.5	0.44	96.5	96.53	3.80	0.356
Error	54	370.0	3.24	1370.0	25.37		
Total	63	21,965.5	100.00				

Second order polynomial model obtained for the hardness is expressed in Eq. (4).

4 $$\begin{aligned} {\text{Hd}} & = - 186.7 + 50.5 A + 0.4630 B + 5.29 C - 0.81 A*A \\ & \quad - 0.000189 B*B - 0.0444 C*C + 0.0260 A*B \\ & \quad + 0.557 A*C - 0.00197 B*C \\ \end{aligned}$$

Hd is hardness, A is the stirring temperature, B the is stirring speed, and C is the stirring time.

ANOVA analysis carried out on impact strength revealed the response is significantly dependent on the input variables (Table 7). Linear terms A, B, and C are significant, squared interactions A*A and C*C are significant while B*B is insignificant. Cross interactions A*B, A*C, and C*C are insignificant on the response. Contributions of A, B, and C are 29.03, 14.87, and 26.50%, therefore, the stirring temperature has the highest contribution. Next is the stirring time while stirring speed contributes the least.

**Table 7:** ANOVA on impact strength (IM).

Source	DF	Seq SS	Contribution (%)	Adj SS	Adj MS	F value	*P* value
A	1	7969.2	29.03	7969.2	7969.19	219.72	0.000
B	1	4355.6	14.87	4833.4	4833.35	133.26	0.000
C	1	7275.8	26.50	8363.6	8363.56	230.59	0.000
A*A	1	4911.6	17.89	4911.6	4911.57	135.42	0.000
B*B	1	460.1	1.68	468.5	468.46	12.92	0.052
C*C	1	1173.4	5.27	1173.4	1173.40	32.35	0.000
A*B	1	70.1	0.26	70.1	70.08	0.26	0.614
A*C	1	611.6	2.23	611.6	611.59	6.35	0.084
B*C	1	9.6	0.03	9.6	9.59	16.86	0.851
Error	17	616.6	2.25	616.6	36.27	1.93	0.182
Total	26	27,453.4	100.00				

Second order polynomial model obtained for hardness is expressed in Eq. (5).

5 $$\begin{aligned} {\text{IM}} & = - 13.15 + 36.1 A + 0.379 B + 3.84 C - 0.5 A*A \\ & \quad - 0.000156 B*B - 0.0313 C*C + 0.0165 A*B \\ & \quad + 0.368 A*C - 0.00102 B*C \\ \end{aligned}$$

IM is the impact strength, A is the stirring temperature, B is the stirring speed, and C is the stirring time.

Table 8 highlights the ANOVA results on the response of elastic modulus to process variables at 95% confidence level and 5% significance level. As reflected, the probability value (p-value) for stirring temperature (A), stirring speed (B), and stirring time (C) as process variables under the linear model is < 0.05 depicting the fact that the parameters contribution to the elastic modulus is significant. However, squared interactions A*A and C*C insignificantly affected the response while B*B is significant. Analysis of the two-way interaction A*B and B*C is insignificant while A*C is statistically significant. Contributions of parameters A, B, and C are 35.16, 17.90, and 25.18% denoting the order of importance of the parameters in descending order of stirring temperature, stirring time, and stirring speed.

**Table 8:** ANOVA for elastic modulus (EM).

Source	DF	Seq SS	Contribution (%)	Adj SS	Adj MS	F value	*P* value
A	1	6993.8	35.16	6993.8	6993.80	276.59	0.000
B	1	5152.1	17.90	5152.0	5152.05	203.75	0.000
C	1	6003.1	25.18	6003.1	6003.11	237.41	0.000
A*A	1	1.0	0.01	1.0	1.00	0.04	0.843
B*B	1	256.3	8.79	256.2	256.25	6.18	0.016
C*C	1	39.1	0.20	39.1	39.06	1.54	0.219
A*B	1	68.1	0.34	68.1	68.06	2.69	0.107
A*C	1	184.6	5.43	184.6	184.64	4.35	0.023
B*C	1	26.0	0.13	26.0	26.01	1.03	0.315
Error	54	136.5	2.87	1365.5	25.29		
Total	63	19,889.4	100.00				

Second order polynomial model was obtained which incorporated the input variable is expressed in Eq. (6).

6 $$\begin{aligned} {\text{EM}} & = - 42.2 + 82.3 A + 1.033 B + 5.86 C - 22.29 A*A \\ & \quad - 0.00571 B*B - 0.0815 C*C + 0.0184 A*B \\ & \quad + 0.643 A*C - 0.00223 B*C \\ \end{aligned}$$

EM is the elastic modulus, A is the stirring temperature, B is the stirring speed, and C is the stirring time.

From Table 9, the *p* values for the linear terms A, B, and C are less than 0.05, hence, they are significant with contributions of 37.80, 7.23, and 29.62% respectively, the relevance of which showed that the input variables have a significant effect on the compressive strength response of the composite. The square terms A*A, B*B, and C*C are significant while the cross interactions A*B, A*C, and B*C are insignificant. From the contributions of the linear terms, the stirring temperature has the highest contribution to the response while the stirring time is the next. Stirring speed showed the least contribution amongst the three parameters. Equation (7) represents the model for compressive strength.

7 $$\begin{aligned} {\text{CS}} & = - 80.9 + 133.8 A + 3.018 B + 24.38 C - 18.81 A*A \\ & \quad - 0.00173 B*B - 0.4381 C*C + 0.055 A*B \\ & \quad + 1.361 A*C - 0.0111 B*C \\ \end{aligned}$$

**Table 9:** ANOVA on compressive strength (CS).

Source	DF	Seq SS	Contribution (%)	Adj SS	Adj MS	F value	*P* value
A	1	69,180	37.80	69,180	69,179.7	375.06	0.000
B	1	13,533	7.23	13,533	13,533.0	69.57	0.000
C	1	60,838	29.62	60,838	60,837.6	293.30	0.000
A*A	1	19,148	10.22	19,148	19,147.6	98.43	0.000
B*B	1	1416	6.76	1416	1415.6	7.28	0.000
C*C	1	7678	5.10	7678	7678.1	39.47	0.009
A*B	1	755	0.40	755	754.9	3.88	0.084
A*C	1	1158	0.62	1158	1157.7	5.95	0.078
B*C	1	3077	1.64	3077	3077.5	15.82	0.060
Error	54	1050	0.61	10,505	194.5		
Total	63	187,286	100.00				

CS is the compressive strength, A is the stirring temperature, B is the stirring speed and C is the stirring time.

### Coefficient of correlation for mechanical properties

Table 10 reveals coefficients of correlation R^2^, R^2^ (adj), R^2^ (pred), and ð R^2^ (difference between R^2^ (adj) and R^2^ (pred)). As for ultimate tensile strength (UTS), R^2^ = 97.75% depicting a strong relationship between the model and the dependent variable that is 97.75% of the observed variation can be explained by the model. R^2^ (adj) is 96.57% while R^2^ (pred) which indicates the level of prediction of future data model is 93.51%. It was reported that the difference (ð R^2^) between R^2^ (adj) and R^2^ (pred) should be < 20% for a reliable model and since the difference is < 20% for UTS there is a good correlation, thus the requirement is satisfied^32–34^. Likewise, the hardness, impact strength, elastic modulus, and compressive strength have a value of R^2^ to be 94.76, 95.9, 96.36, and 97.39 showing that the model has over 90% representation of the relationships. ð R^2^ (%) for hardness, impact strength, elastic modulus and compressive strength are all less than 20% hence satisfying the requirement and said to have good correlation.

**Table 10:** Coefficient of correlation.

Parameters	R^2^ (%)	R^2^ (adj) (%)	R^2^ (pred) (%)	ð R^2^ (%)
Ultimate tensile strength	97.75	96.57	93.51	< 20
Hardness	94.76	93.72	91.83	< 20
Impact strength	95.90	94.54	92.02	< 20
Elastic modulus	96.36	95.42	93.74	< 20
Compressive strength	97.39	96.46	94.18	< 20

### Normal probability plots and residual versus fit

**Figure 1:**
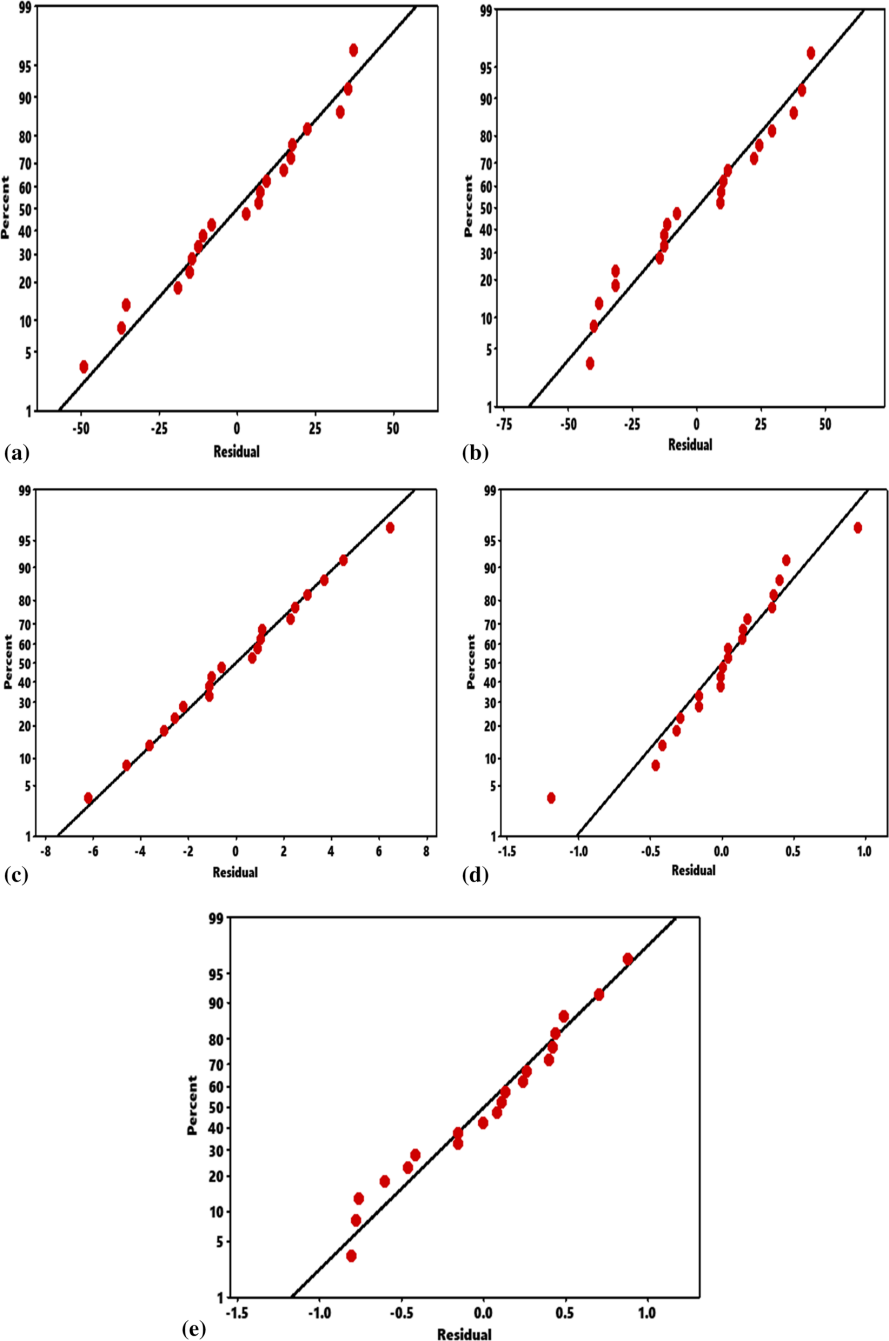
Normal probability plot for responses. (**a**) Ultimate tensile strength, (**b**) hardness, (**c**) impact strength, (**d**) elastic modulus, (**e**) compressive strength.

**Figure 2:**
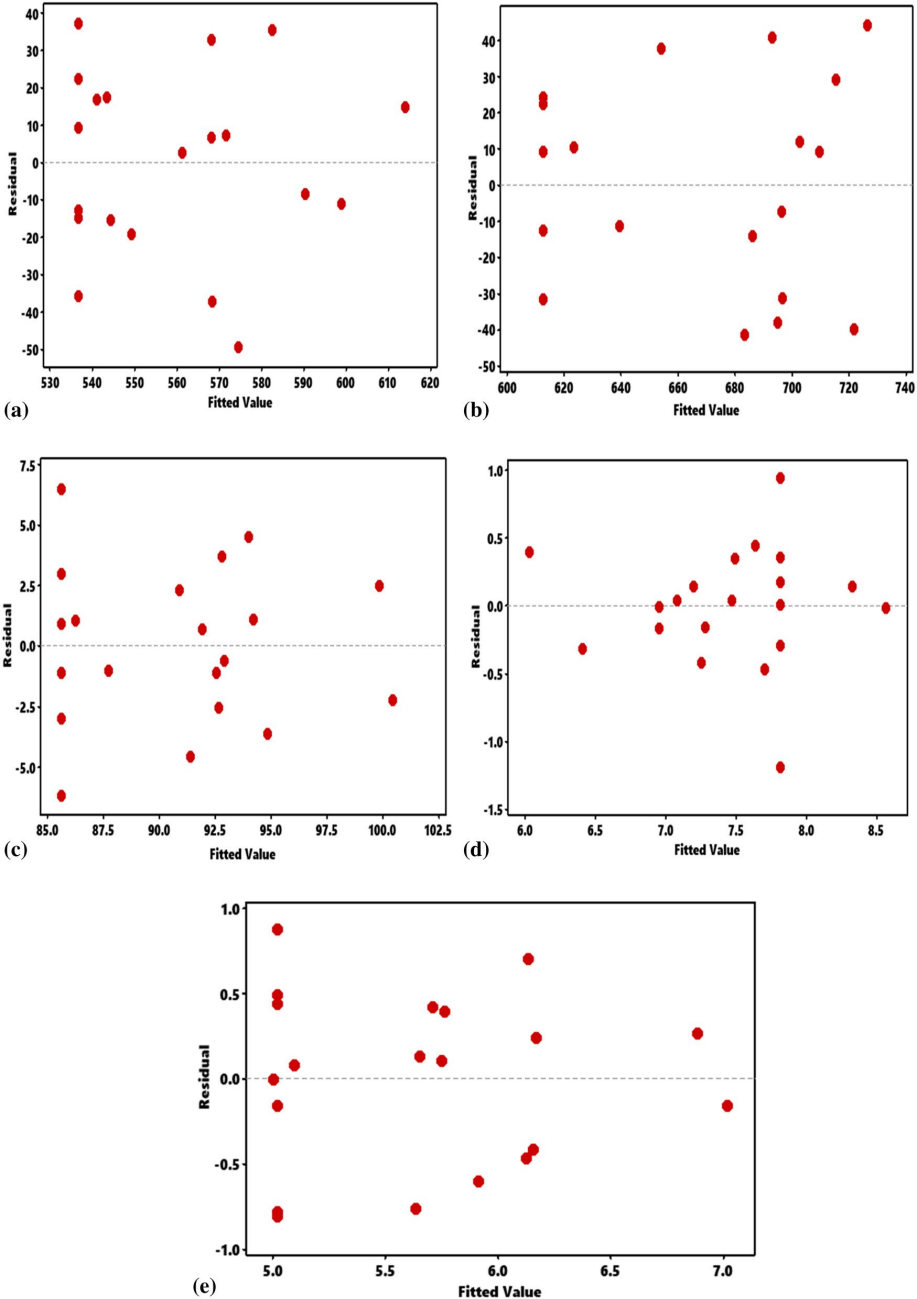
Normal probability plot of residuals for responses. (**a**) Ultimate tensile strength, (**b**) hardness, (**c**) impact strength, (**d**) elastic modulus, (**e**) compressive strength.

Figure 1a–e illustrates the normal probability plot of residuals for ultimate tensile strength, hardness, impact strength, elastic modulus, and compressive strength. It is observed that most of the plotted points form a nearly linear pattern with few departures from the straight line showing consistent data, further confirming the validity of the model. Scatter plot of the residual against fitted data is demonstrated in Fig. 2a–e in which the data are plotted randomly round the dashed line is heteroskedastic as the points are concentrated towards the line^33–35^. The residual for the responses is uniformly scattered around the mean response, reflecting the adequacy of the model.

### Analysis of the response surface and contour plot

#### Response surface and contour plot for ultimate tensile strength

##### Effect of interaction of stirring temperature (Tm) versus stirring speed (Sp) on the ultimate tensile strength of composite

**Figure 3:**
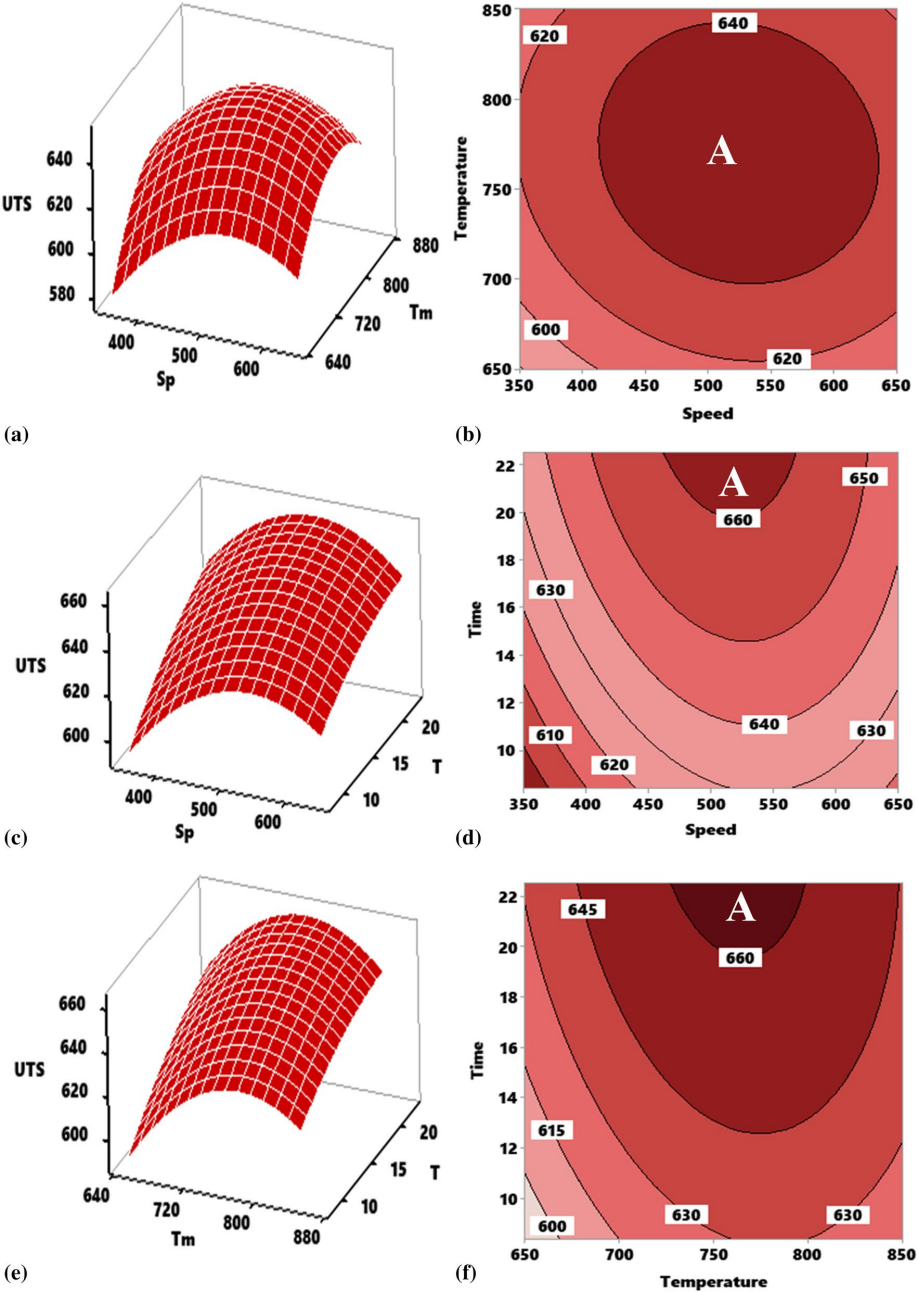
Influence of stirring parameter on ultimate tensile strength of developed composite as represented by response surface plot and contour plot for interactions. (**a**, **b**) Speed and temperature, (**c**, **d**) speed and time, (**e**, **f**) temperature and time.

Property responses as a function of interactions between experimental variables are represented by a 3D response surface plots and 2D contour mapping. The graphical illustration of the model is plotted as a function of two process parameters holding the other variable constant. As for the response surface, the response is plotted on Z axis while the input variables are plotted on X and Y axis. The effect of interaction between speed and temperature at a constant time of 15 min is highlighted in Fig. 3a for the response surface. As the stirring speed, temperature, and speed increased, ultimate tensile strength response increased. However, at the speed of 500 rpm, and a temperature of 800 °C, the ultimate tensile strength response value reduced. There is therefore a strong dependence of the ultimate tensile strength on the interactive pattern between speed and temperature. The two parameters displaced a parabolic reflection profile with points of inflection at 800 °C for temperature and 500 rpm for speed, yielding a maximum value of 644.8 MPa at the point of inflexion. Figure 3b—shows the contour plot of the effect of the interaction temperature versus speed at constant 15 min on UTS. The plot revealed different portions where varying ultimate tensile strength can be attained at varying temperature and speed. Portion A is the optimal zone in which the interaction of temperature and speed yield an optimum strength range of 640–660 MPa attainable at a temperature range of 720–841 °C and speed of 440–635 rpm.

##### Effect of interaction of stirring time (T) versus stirring speed (Sp) on the ultimate tensile strength of composite

The interactive effect of stirring time and speed at a constant stirring temperature of 700 °C is illustrated in Fig. 3c. As the stirring time and speed simultaneously increased, there was an improvement in strength, however, there was a significant negative interaction between speed and temperature at speed beyond 500 rpm, the effect of which led to a decrease in strength. The increase in strength is traceable to adequate speed and stirring time aiding the dispersion of particles within the melt. Increasing speed beyond 500 rpm ensued high turbulence during stirring leading to gas entrapment and blow holes causing a decrease in strength. Stirring speed showed a parabolic profile while time demonstrated a linear profile with positive gradient. The contour plot of the two parameters is elucidated in Fig. 3d, where portions are segmented by boundary line. The plot shows that an optimum values for strength can be realized a portion designated as A which is the optimum portion where the strength range of 660–675 MPa can be attained by the combined interplay of stirring speed in the range 462–555 rpm and stirring time between 20 and 22.5 min.

##### Effect of interaction of stirring time (T) versus stirring temperature (Tm) on ultimate tensile strength of composite

Plot of Fig. 3e shows the result of the interactive effect of time and temperature on the ultimate tensile strength of the developed aluminum composite at constant stirring speed 500 rpm. Increasing temperature and time led to an improvement in the ultimate tensile strength of which at temperature beyond 750 °C there is a negative interaction between temperature and time effect leading to a decrease in strength. Therefore, the profile of stirring temperature is parabolic with a point of inflection at 750 °C, while the stirring time reflects a linear interaction profile of positive gradient. Interaction of the two parameters yielded a maximum strength of 665.8 MPa at 750 °C and 22.5 min.

Contour plot of the interaction time versus temperature as it affects the ultimate tensile strength is made available in Fig. 3f. As observed, increasing time and temperature led to the enhancement of strength. Portion designated “A” pinpointed the region of attaining optimum strength which covers the strength range of 660–675 MPa at a temperature of 735–800 °C and duration of 19.4–22.5 min.

#### Response surface and contour mapping for microhardness

##### Effect of interaction of stirring temperature (Tm) versus stirring speed (Sp) on Vickers microhardness of composite

**Figure 4:**
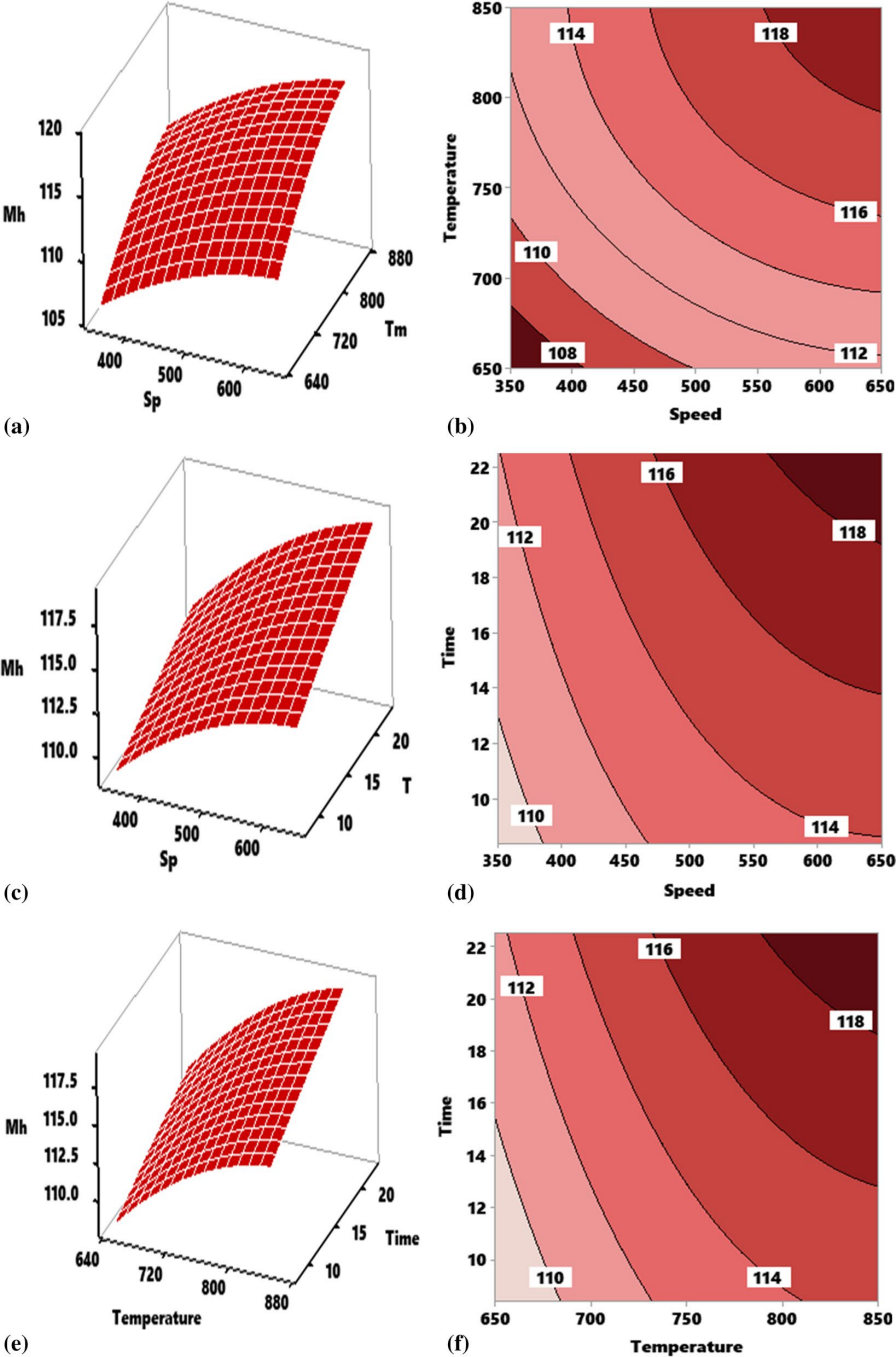
Influence of stirring parameter on microhardness of developed composite as represented by response surface plot and contour plot for interactions. (**a**, **b**) Speed and temperature, (**c**, **d**) speed and time, (**e**, **f**) temperature and time.

Figure 4a shows the effect of the interaction between speed and temperature on Vickers micro hardness of the developed aluminum composite reinforced with 10 wt% TiO_2_ ceramic microparticles at a constant time of 15 min. With the increase in speed and temperature from -1 level, there was a progressive enhancement of the hardness. This shows that the hardness of the composite depends on the interaction between the two parameters. The two input variables of speed and temperature reflected a concave profile as observed in the figure. Maximum hardness of 119.2 HV was attained at an interplay of 600 rpm and 835 °C. The figure further shows that the response of microhardness depends on the interaction. Contour plot as indicated in Fig. 4b show the regions in which varying values of hardness are attained. Portion A highlights the region of the plot where maximum microhardness > 118 is attained at a stirring speed range of 562–650 rpm and stirring temperature of 800–850 °C.

##### Effect of interaction of stirring time (T) versus stirring speed (Sp) on Vickers microhardness of composite

The response of hardness to the interaction between speed and time holding the temperature constant at 750 °C is presented in Fig. 4c. Increasing speed and time resulted in the enhancement of hardness. Stirring speed presented a concave profile while the stirring time had a linear profile with a positive gradient. The figure reflects that the microhardness of the composite depends on the interaction between the two parameters. Maximum microhardness of 119.2 HV was attained when the process was carried out at 580 rpm for 20 min. The plot of Fig. 4d shows that the optimum hardness can be attained at the portion “A” value of which is between 118 and 120 HV. Corresponding range of speed is 560–650 rpm while that of time is 19.4–22.5 min.

##### Effect of interaction of stirring time (T) versus stirring temperature (Tm) on Vickers microhardness of composite

From Fig. 4e, the effect of the interaction of stirring time and the temperature on Vickers microhardness of the developed aluminum composite reinforced with TiO_2_ ceramic microparticles at a constant speed of 500 rpm. With the increase in temperature and time, there was a consecutive increase in hardness. This shows that the hardness of the composite depends on the interaction between the two parameters. Temperature reflected a concave profile while time depicted an upward linear profile. Maximum hardness of 119.2 was attained at the interplay of 600 rpm and 835 °C. The figure further shows that the response of microhardness depends on the interaction. The plot of Fig. 4f shows that the optimum hardness can be attained at the portion “A” value of which is 118–120 HV. Corresponding range of speed is 785–850 °C while that of time is 16.5–22.5 min.

#### Response surface and contour mapping for impact strength

##### Effect of interaction of stirring temperature (Tm) versus stirring speed (Sp) on the impact strength of composite

**Figure 5:**
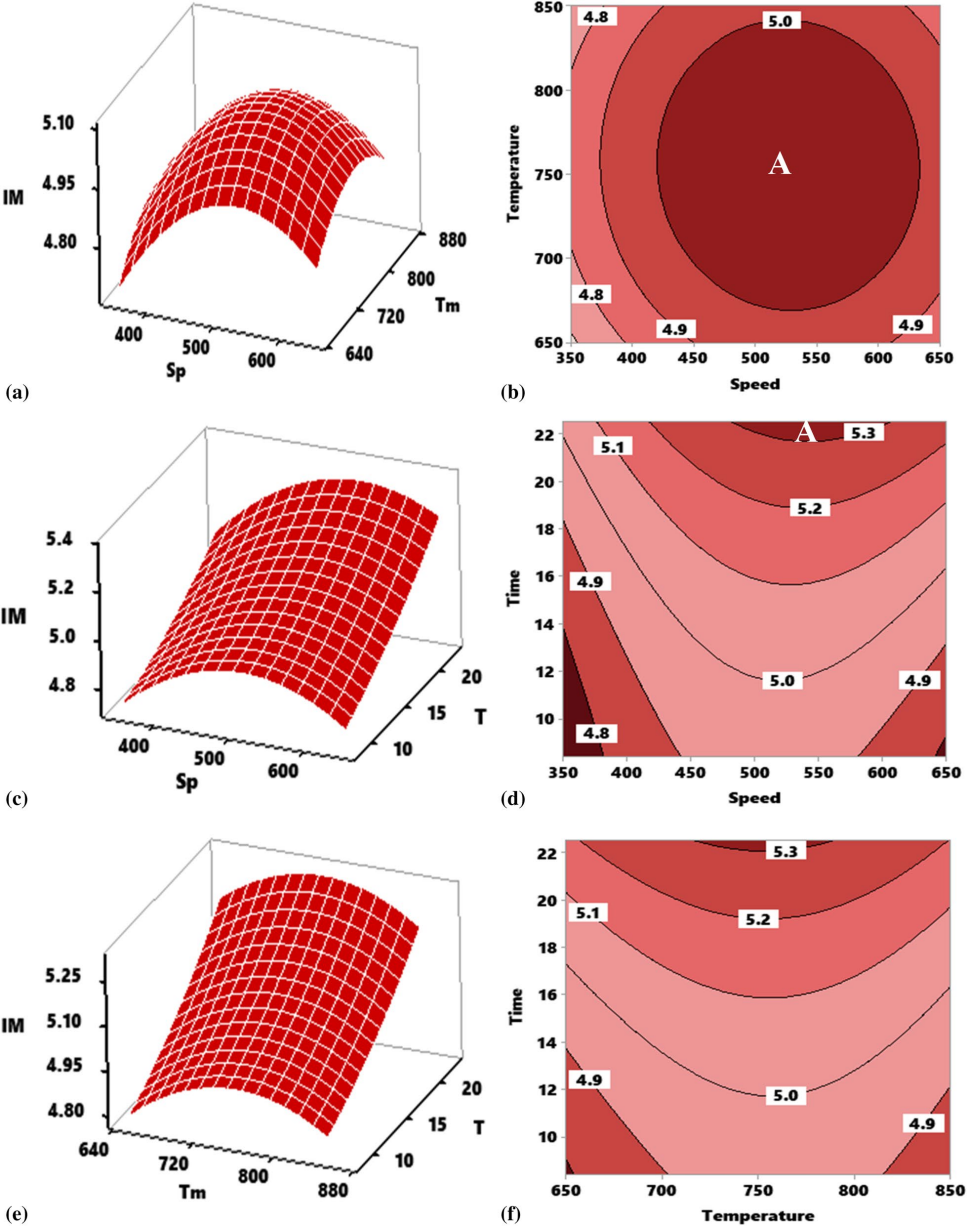
Influence of stirring parameter on impact strength of developed composite as represented by response surface plot and contour plot for interactions. (**a**, **b**) Speed and temperature, (**c**, **d**) speed and time, (**e**, **f**) temperature and time.

The effect of interaction between speed and temperature at a constant time of 15 min is presented in Fig. 5a for the response surface. As the stirring speed, temperature, and speed increased, the impact strength response increased, nonetheless, at the stirring speed of 500 rpm, and a temperature of 800 °C, the impact strength response value decreased. There is therefore a strong dependence of impact strength on the interactive pattern between speed and temperature. The two experimental parameters displaced a parabolic profile with the ends facing down.

The points of inflection at 800 °C for temperature and 500 rpm for speed yields a maximum value of 5.27 J/m^2^. Figure 5b displays the contour plot of the effect of the interaction temperature versus speed at constant 15 min on impact strength. The plot shows different portions where varying impact strength can be realized at different temperature and speed interactions. Portion “A” is the optimal zone in which the interaction of temperature and speed bore an optimum strength beyond 5.0 MPa realizable at a temperature range of 675–842 °C and speed of 440–635 rpm.

##### Effect of interaction of stirring time (T) versus stirring speed (Sp) on the impact strength of composite

The interactive effect of stirring time and speed at a constant stirring temperature of 700 °C is highlighted in Fig. 5c. As the stirring time and speed simultaneously increased, there was an improvement in strength, however, there was a significant negative interaction between speed and temperature at speed beyond 500 rpm, the effect of which ensued a strength decrease. Accretion in strength can be associated with adequate speed and stirring time, aiding the uniform dispersion of particles within the melt. Increasing speed beyond 500 rpm on the other hand, causes turbulence during stirring leading to gas entrapment and blow holes causing a decrease in strength. Stirring speed showed a parabolic profile with the two ends tending downwards, while time demonstrated an upward linear profile with positive gradient. Contour plots of the two parameters as depicted in Fig. 5d revealed portions segmented by a boundary lines. Optimum impact strength is realizable at a portion designated ‘A’ in which a strength range of 5.3–5.4 J/m^2^ is attainable by the combined interplay of stirring speed at 480–600 rpm and a stirring time of 21.4–25 min.

##### Effect of interaction of stirring time (T) versus stirring temperature (Tm) on the impact strength of composite

Plot of Fig. 5e reveals the result of the interactive effect of time and temperature on the impact resistance of the developed aluminum composite at constant stirring speed 500 rpm. Increasing temperature and time amounted to an improvement in impact strength of which at temperature beyond 750 °C there is a negative interaction between temperature and time effect leading to a decrease in strength. Therefore, the profile of stirring temperature is parabolic with a point of inflection at 750 °C, while the stirring time reflects a linear interaction profile of positive gradient. Interaction of the two parameters yielded a maximum strength of 5.32 J/m^2^ at 750 °C and 22.5 min. Figure 5f highlights the contour plot of the interaction time versus temperature as it affects the impact strength. As demonstrated, increasing time and temperature led to the enhancement of impact strength. Portion designated “A” pinpointed the region of attaining optimum strength which covers the strength range of 5.3–5.4 J/m^2^ at a temperature of 725–785 °C and duration of 21.5–22.5 min.

#### Response surface and contour mapping for elastic modulus

##### Effect of interaction of stirring temperature (Tm) versus stirring speed (Sp) on elastic modulus of composite

**Figure 6:**
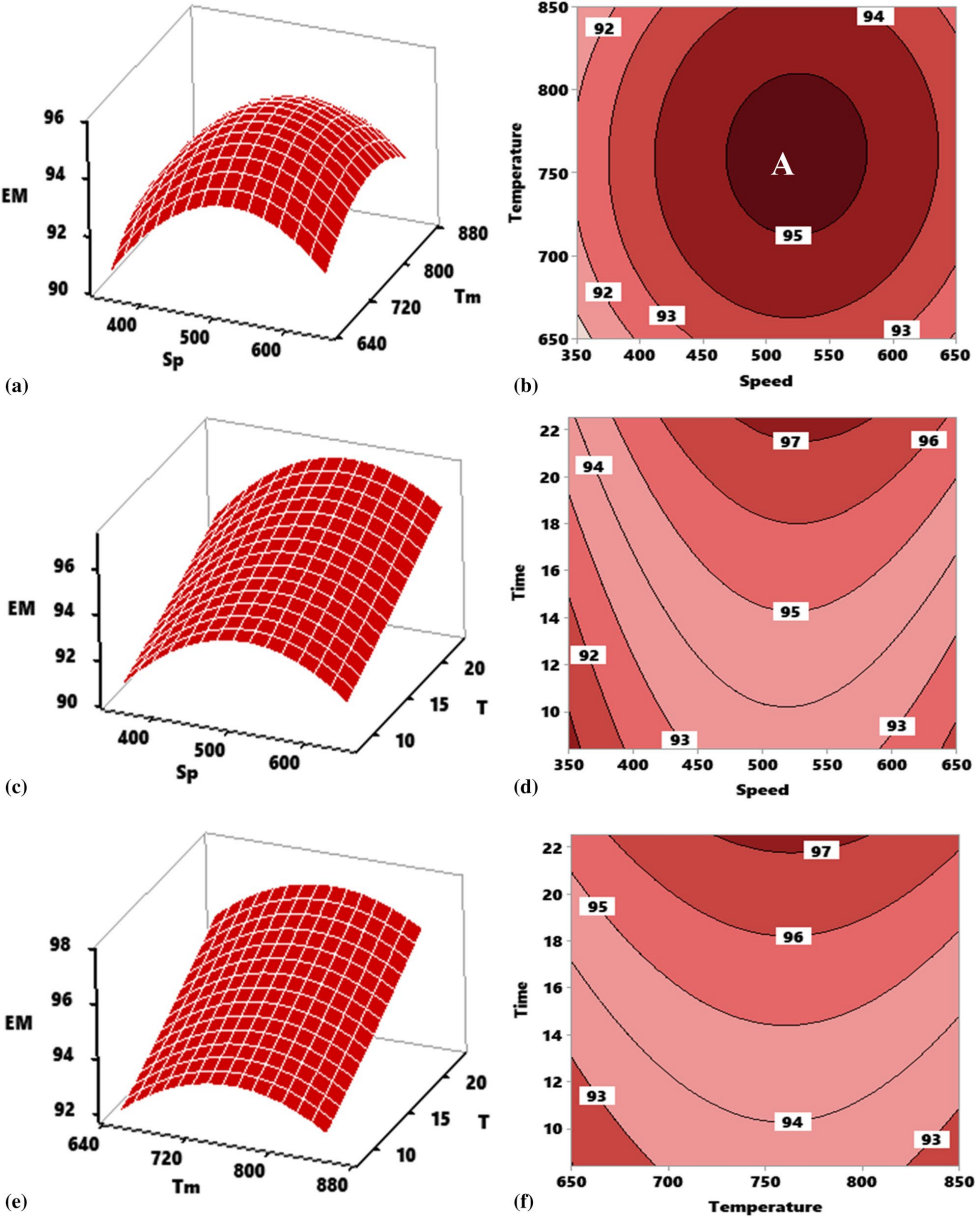
Influence of stirring parameter on elastic modulus of developed composite as represented by response surface plot and contour plot for interactions. (**a**, **b**) Speed and temperature, (**c**, **d**) speed and time, (**e**, **f**) temperature and time.

The influence of interaction between speed and temperature at a constant time of 15 min is highlighted in Fig. 6a for the response surface. As the stirring speed, temperature, and speed increased, the elastic modulus increased, although an interplay between the speed of 500 rpm and a temperature of 750 °C engendered decrease in the modulus. There is a strong dependence of the ultimate tensile strength on the interactive pattern between speed and temperature. The two parameters displaced a parabolic interactive profile with points of inflection at 750 °C for temperature and 500 rpm for speed with a maximum value of 95.5 GPa at the point of inflexion. Figure 6b shows the contour plot of the effect of the interaction temperature versus speed at constant 15 min on the elastic modulus. The plot reflects different segments where varying elastic moduli are attained at varying temperature and speed interaction. Segment “A” is the optimal zone in which the interaction of temperature and speed yield an optimum strength range of 94–96 GPa attainable at a temperature range of 720–824 °C and speed of 475–580 rpm.

##### Effect of interaction of stirring time (T) versus stirring speed (Sp) on the elastic modulus of composite

The interactive effect of stirring time and speed at a constant stirring temperature of 700 °C is depicted in Fig. 6c. As the stirring time and speed simultaneously increased, there was improvement in the modulus. Notwithstanding, there was a significant negative interaction between speed and temperature at speed beyond 500 rpm, the effect of which led to decrease in the modulus. Stirring speed showed a parabolic profile with the two-end pointing downwards while time demonstrated a linear uptrend profile. The contour plot of the two parameters is presented in Fig. 6d where the portions are segmented by the boundary lines. The plot shows that the optimum values for strength can be realized at the portion designated ‘A’ region in which a modulus of the range 97–98 GPa can be attained by the combined interaction of stirring speed between 470 and 585 rpm and stirring time between 18.7 and 22.5 min.

##### Effect of interaction of stirring time (T) versus stirring temperature (Tm) on the elastic modulus of composite

Plot of Fig. 6e presents the result of the interactive effect of time and temperature on the elastic modulus of the developed aluminum composite at constant stirring speed 500 rpm. As temperature and time increased, there was an increment in the elastic modulus of which at temperature beyond 750 °C there is a negative interaction between temperature and time effect leading to a decrease in the modulus. The profile of stirring temperature is parabolic with the point of inflection at 750 °C, while the stirring times reflects a linear interaction profile of positive gradient. Interaction of the two parameters yielded a maximum elastic modulus of 97.2 GPa at 750 °C and 22.5 min. Contour plot of the interaction time versus temperature as it affects the elastic modulus is made represented in Fig. 6f. As observed, increasing time and temperature led to enhancement of the modulus. Portion designated “A” pinpoints the region of attaining the optimum modulus in the range of 97–98 GPa at a temperature of 720–816 °C and duration of 19.5–22.5 min.

#### Response surface and contour mapping for compressive strength

##### Effect of interaction of stirring temperature (Tm) versus stirring speed (Sp) on compressive strength of composite

**Figure 7:**
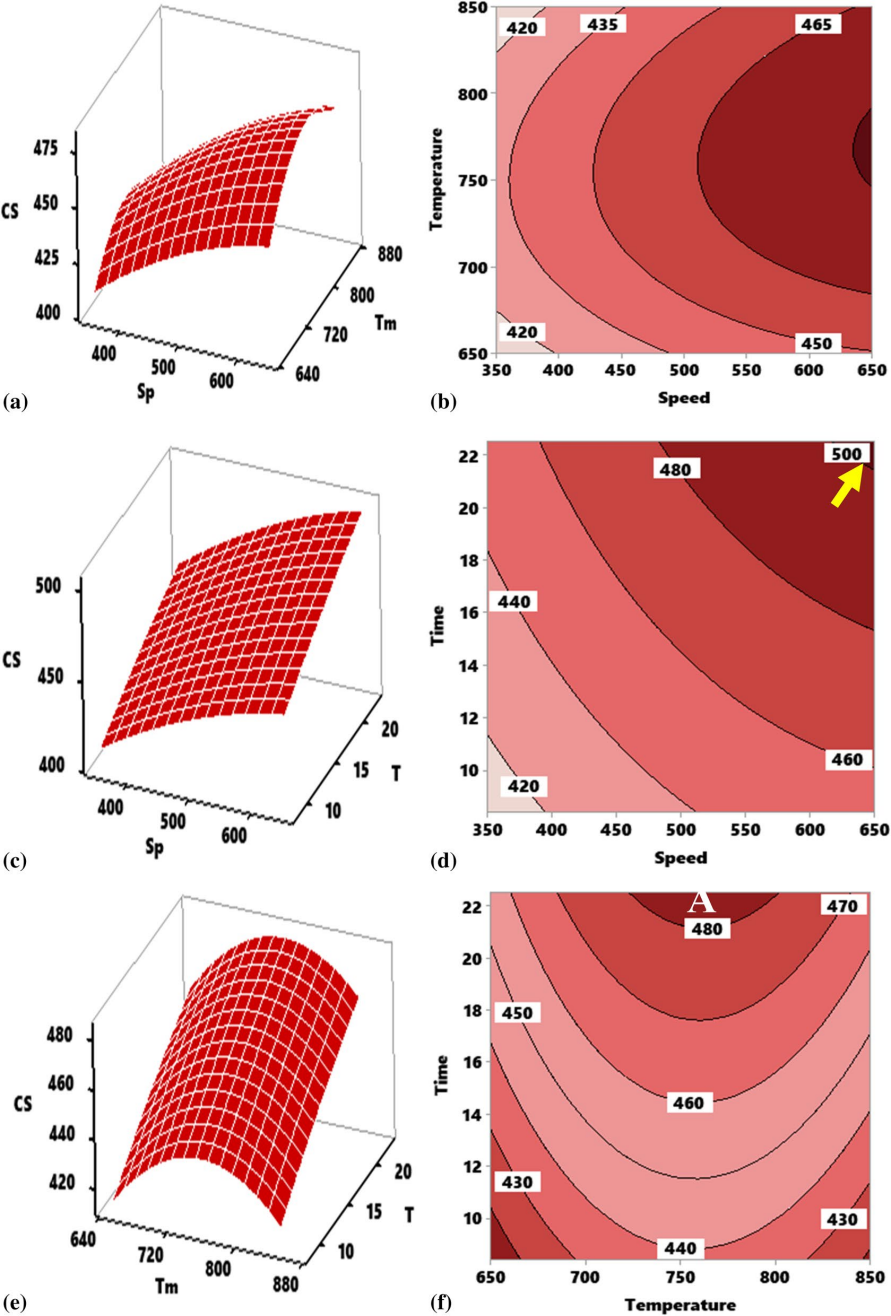
Influence of stirring parameter on compressive strength of developed composite as represented by response surface plot and contour plot for interactions. (**a**, **b**) Speed and temperature, (**c**, **d**) speed and time, (**e**, **f**) temperature and time.

Figure 7a shows the effect of the interaction between speed and temperature on the compressive strength of the developed aluminum composite reinforced with 10 wt% TiO_2_ ceramic microparticles at a constant time of 15 min. With an increase in speed and temperature, there was a progressive rise in compressive strength. Therefore, the strength response depends on the interaction. The two input variables of speed and temperature reflected a concave profile as observed in the figure. Maximum strength of 484.3 MPa was attained at an interplay of 650 rpm and 820 °C. Contour plot as indicated in Fig. 7b show regions in which varying values of compressive strength 480–495 MPa were attained at the stirring temperature of 742–792 °C and 630–650 rpm. The maximum hardness is higher than that of the reference material by 38.7%.

##### Effect of interaction of stirring time (T) versus stirring speed (Sp) on the compressive strength of composite

Compressive strength response as regards the interaction between speed and time holding temperature constant at 750 °C is presented in Fig. 7c. Increasing speed and time resulted in enhancement of the strength. Stirring speed presented a half parabola while the stirring time had a linear profile with a positive gradient. The figure reflects that the compressive strength of the composite depends on the interaction between the two parameters. Maximum strength of 514.8 MPa was attained at 650 rpm for 22.5 min. The plot of Fig. 7d shows that the optimum strength can be attained at a portion A, value of which is 500–520 MPa. Corresponding range of speed is 642–650 rpm while that of time is 21.5–22.5 min.

##### Effect of interaction of stirring time (T) versus stirring Temperature (Tm) on the compressive strength of composite

From Fig. 7e, the effect of the interaction of stirring time and Temperature on the compressive strength of the developed aluminum composites reinforced with TiO_2_ ceramic microparticles at a constant speed of 500 rpm was evident. With an increase in temperature, and time there was a progressive rise in compressive strength (Fig. 7f). This shows that the strength of the composite depends on the interaction between the two parameters. The temperature reflected a parabolic profile with the two ends facing downward, while time depicted an upward linear profile. A maximum strength of 486.8 MPa was attained at an interplay of 650 rpm and 22.5 min. The figure further shows that the response of compressive strength depends on the interaction.

### Optimization and model validation

With the use of Minitab 19, the response surface method was employed in the optimization of the factors; stirring temperature, speed, and time. The independent factors were ranged from lower to upper limits and the tensile strength was maximized. Optimization was done with no constraint at 95% confidence level while lower and upper levels were fixed for all parameters. Optimal values were obtained for the ultimate tensile strength, microhardness, impact strength, elastic modulus, and compressive strength fit at desirability of 0.91 as indicated in Table 11. The optimum conditions for stirring are 574.2 rpm, 779.3 °C, and 22.5 min for speed, temperature, and time for optimum tensile overall properties respectively. Validation of experimental run were done applying the optimum experimental inputs at 574.2 rpm, 779.3 °C, and 22.5 min for speed, temperature, and time, respectively. Average experimental results obtained are presented in Table 11. It is evident that the deviation for each property is less than 5%, therefore, it is concluded that there is a good agreement between the experimental values and the predicted value, thus, validating the model.

**Table 11:** Optimal parameters and validation result.

Optimal parameters			
Temperature	Speed	Time	
779.3 °C	574.2 rpm	22.5 min	

	Predicted values	Experimental value	Deviation (%)
Compressive strength	495.1 MPa	487.7 MPa	1.49
Elastic modulus	97.1 GPa	99.8 GPa	2.78
Impact strength	5.3 kJ/m^2^	5.53 kJ/m^2^	4.34
Microhardness	119.3 HV	116.8 HV	2.10
Ultimate tensile strength	658.4 MPa	641.3 MPa	2.60

Composite desirability = 0.94.

### Microstructural and XRD analysis

**Figure 8:**
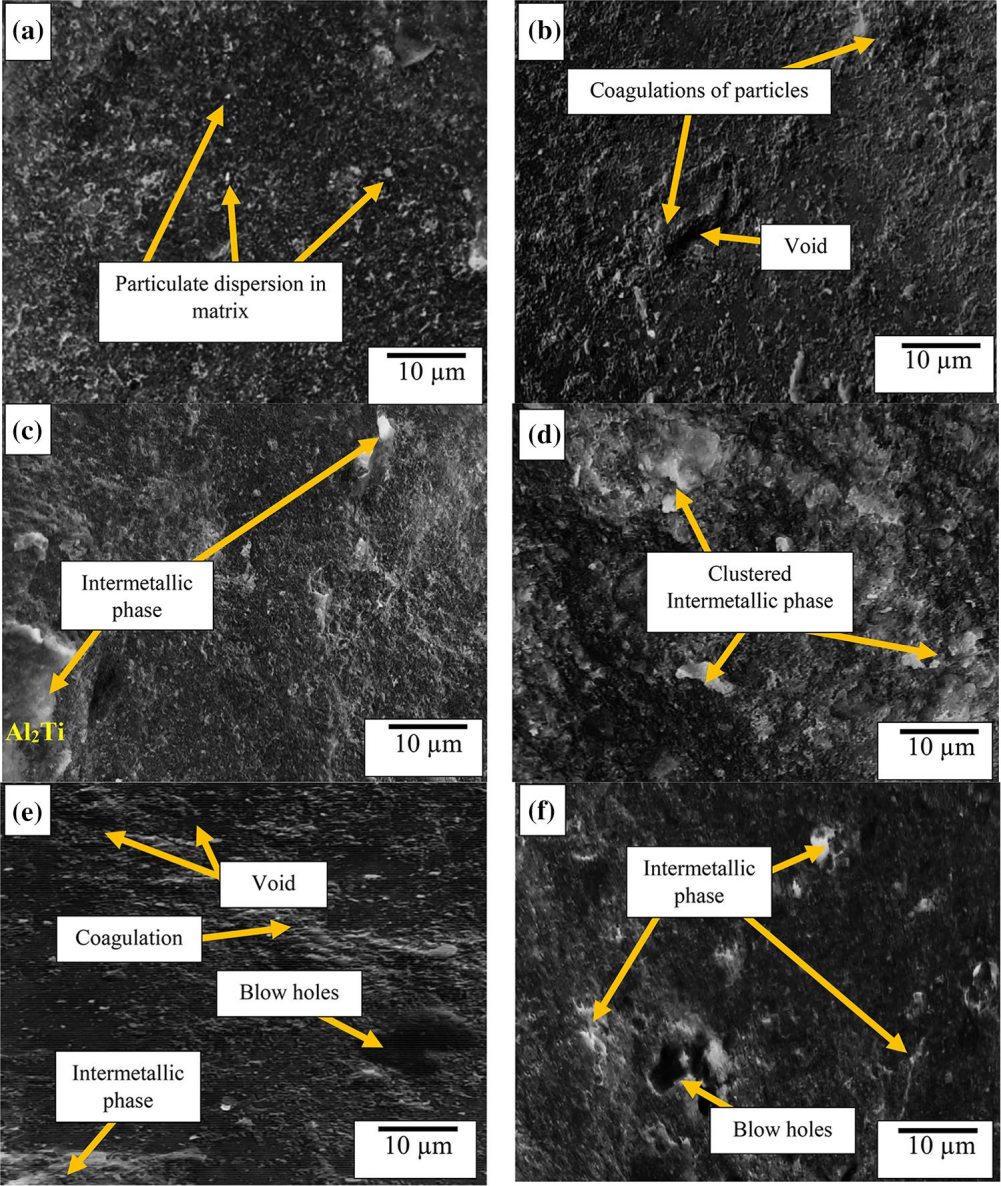
Microstructural images of developed composite showing different conditions of experimental runs at temperature, speed, and time values of (**a**) 700 °C, 500 rpm, 15 min; (**b**) 350 °C, 331.821 rpm, 10 min; (**c**) 868.179 °C, 500 rpm, 15 min; (**d**) 800 °C, 600 rpm, 20 min; (**e**) 800 °C, 600 rpm, 10 min; (**f**) 800 °C, 600 rpm, 20 min.

Figure 8a–f show the microstructural image of samples produced at different conditions according to the dictated experimental runs. Figure 8a reflects the uniform dispersion of particulates within the matrix for the composite produced under the experimental conditions of 500 rpm, 700 °C, and 15 min. Figure 8b reflects the dispersion of particles and coagulation, which is attributed to the samples prepared at low temperature of 350 °C, speed of 331.821 rpm and time 10 min. At lower temperature, the viscosity is higher which has the tendency for premature solidification leading to coagulation^36^. Figure 8c and d features intermetallic phases which occurred at high temperature of 868.179 °C leading to the formation of intermetallic phases culminating in the lowering of strength. Meanwhile, the moderate temperature allows the mobility of atoms allowing the TiO_2_ to be dispersed within the matrix.

Figure 8 e and f are images of samples produced at temperature and speed of 800 °C and 600 rpm, respectively. At high temperature and speed, there is turbulence during stirring leading to gas entrapment causing voids and blow holes as observed in the figures^37^.

**Figure 9:**
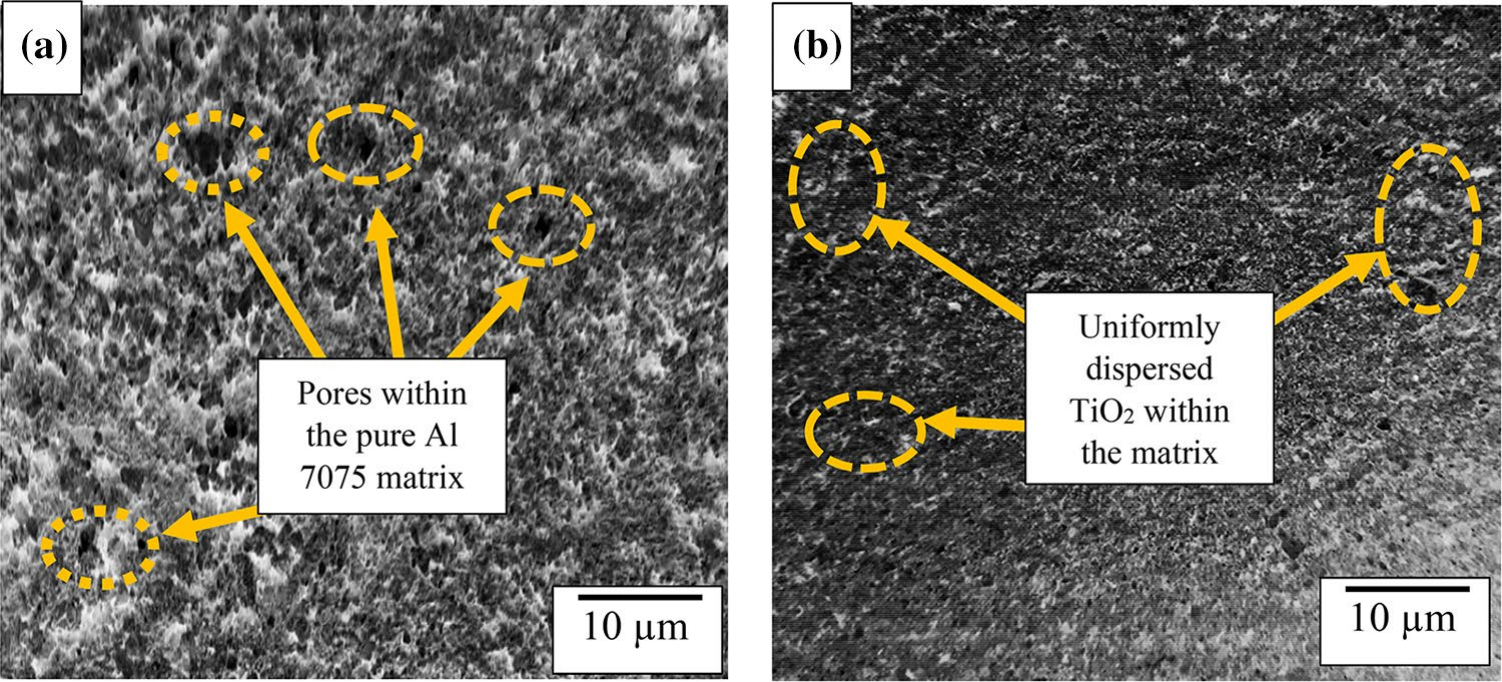
Microstructural image of (**a**) pure al 7075 and (**b**) developed composite at optimal stirring parameters.

Figure 9a presents the microstructural image of the pure aluminum base alloy in which inherent pores are detected. Figure 9b showed the morphology of the developed composites at optimum stirring parameters of 779.3 °C, 574.2 rpm, 22.5 min for temperature, speed, and time, respectively. The image showed uniform dispersion of the particulate within the matrix giving rise to enhanced properties as compared to the pure Al 7075 alloy.

**Figure 10:**
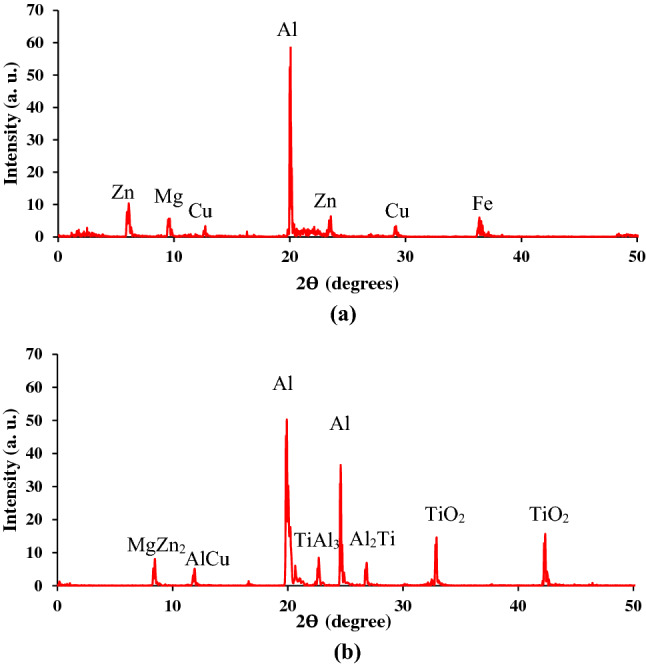
XRD patterns in (**a**) pure Al 7075 and (**b**) composite developed at optimal stirring parameters.

Figure 10a and b indicates the phases present in the pure Al 7075 alloy and the developed composite at optimal stirring parameters. In Fig. 10a, the XRD patterns of the pure alloy showed the presence of crystalline aluminum and traces of other metal components. The patterns in Fig. 10b identified other phases alongside the crystalline aluminum. Titanium dioxide is present confirming the presence of particulate which suppresses the existence of other trace elements identified in the base metal. Traces of intermetallic phases are also identified, TiAl_3_ and Al_5_T_12_ which occurred as a result of high temperature reaction. Since in trace, they are suppressed, therefore not playing a major role on the properties of the composite.

## Conclusion

Influence of individual and combined interaction of three processing factors of stir casting processing route in the development of Al 7075/TiO_2_ composite was evaluated by means of the central composite design using the response surface method. Outcome of the findings showed that the response of ultimate tensile strength, hardness, impact strength, elastic modulus, and compressive strength depends on the interactions between these parameters. The ANOVA results showed that each property is influenced by the stirring parameters while selected square interactions had considerable effect at confidence level of 95%. Five predictive models were developed for the properties were observed to be statistically significant and from the coefficient of correlation, it was deduced that over 94% of the data for each response was well represented by the model. Response surface method revealed that each evaluated has a strong dependence on the interactions between the process variables. Parameter values not greater than 750 °C and 500 rpm for temperature and speed, respectively, were observed to have a positive influence on the responses, while values beyond that could have negative contributions. In the same manner, a stirring time of up to 30 min may provide enough time for even dispersion engendering a positive contribution to the responses. Simultaneous optimization of the properties showed that the optimum of mechanical properties is achievable at experimental conditions of 779.3 °C, 574.2 rpm, and 22.5 min.

## Data Availability

All data generated or analyzed during this study are included in this published article.

## Code availability

No code was used for the computation of the data reported in this study.
